# Treatment experience of thoracic aortic aneurysm recurrence after multiple surgeries for Marfan syndrome: A case report

**DOI:** 10.1097/MD.0000000000017391

**Published:** 2019-10-04

**Authors:** Huai-Dong Chen, Fan He, Xi-Ming Qian

**Affiliations:** Department of Cardiac Surgery, Sir Run Run Shaw Hospital, School of Medicine, Zhejiang University, Hangzhou, China.

**Keywords:** aortic aneurysm, Marfan syndrome, recurrence, TEVAR

## Abstract

**Rationale:**

Marfan syndrome (MFS), an autosomal dominant hereditary disease, often results in structural and functional abnormalities of the aortic wall. Because of residual aortic aneurysm or aortic dissection, patients with MFS usually need repeat operations after the first operation.

**Patient concerns:**

A patient diagnosed with MFS who had undergone 2 surgeries because of abdominal aortic dissection aneurysm and Stanford A type aortic dissection at different times.

**Diagnoses:**

MFS.

**Interventions:**

Due to aneurysmal dilatation of the residual descending aorta, we performed the third surgery for him through using 2 artificial stent grafts to fix and package the area of the remaining vessel in our hospital.

**Outcomes:**

The result was good, and the patient was discharged successfully.

**Lessons:**

Using artificial material to repair and wrap the area of the residual vessel during the first surgery can provide an anchor area for future endovascular stent implantation and also offer a possibility for stent implantation in patients with MFS.

## 1 Introduction

Marfan syndrome (MFS), an autosomal dominant hereditary disease, often results in structural and functional abnormalities of the aortic wall, such as aortic aneurysm enlargement and aortic dissection.^[1]^ The most severe complication of MFS is aortic aneurysm leading to aortic dissection, rupture, and death.^[1]^ Current evidence suggests that median sternotomy is the ideal setting in these patients.^[2]^ whereas, even after successfully surgical dissection repair, the residual aorta and the presence of the aortic false lumen are confronted with an ongoing risk for aneurysm formation, reoperation, or sudden death.^[3,4]^ At present, endovascular treatment can significantly reduce morbidity and mortality, including high-risk patients with complicated type B dissection.^[5]^ But for MFS, there is still a lot of controversies. Thoracic endovascular aortic repair (TEVAR) has long been considered a relative contraindication for MFS patients.^[6]^ And small series of reports about TEVAR in the patients of MFS have also been published,^[6–11]^ However, feasibility and safety of TEVAR for the patients with residual aortic dissection after multiple operations remains unclear. Here we report a patient with MFS and share our treatment experience of thoracic aortic aneurysm recurrence after multiple surgeries.

## 2 Case report

**Figure 1:**
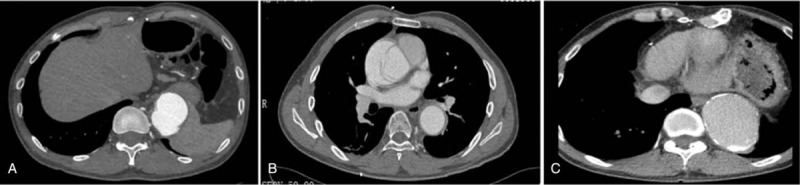
A: The first CTA (2005) revealed an abdominal aortic aneurysm dissection. B: The second CTA (2012) revealed a sign of Stanford type A aortic dissection. C: The third CTA revealed an aneurysmal dilatation of the descending aorta.

A 35 years old male patient had undergone the operation of abdominal aorta artificial vascular replacement because of MFS and abdominal aortic dissection aneurysm in March 2005 (Fig. 1A). In December 2012, he had experienced sudden, severe chest pain and was readmitted to our hospital. The aortic contrast-enhanced computed tomography (CT) showed a sign of Stanford A type aortic dissection (Fig. 1B). The echocardiography revealed aortic sinus expansion with moderate or severe aortic valve insufficiency. The patient underwent second operation of aortic valve replacement, ascending aortic replacement and descending aorta-nasal stent implantation and was discharged on the seventh postoperative day. The patient came back to our hospital for a follow-up with aorta computed tomography angiography (CTA) on September 4th, 2018. The CTA revealed that the descending aorta has an aneurysmal dilatation for a 12.4 cm length. The cross-section diameter of the widest part was about 74 mm × 55 mm (Fig. 1C). The patient was hospitalised for the third time and was diagnosed with thoracic aortic aneurysm.

**Figure 2:**
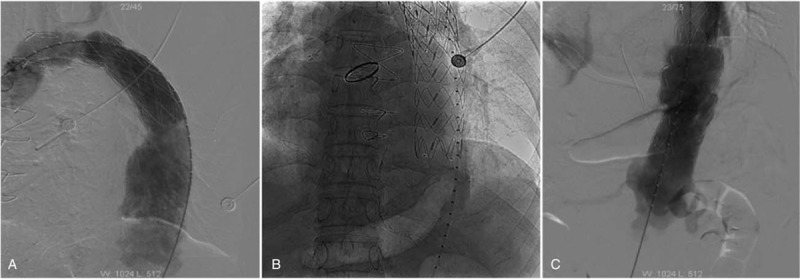
A: The first stent was inserted with the proximal end anchored firmly. B: The second stent was inserted and well positioned. C: Two stents were inserted successfully, and angiography demonstrated that the thoracic aortic aneurysm had disappeared and had no internal leakage.

On September 11th, 2018, the patient underwent his third operation of TEVAR, we chose the transfemoral approach and inserted 2 consecutive Medtronic endovascular stent grafts (42 × 150 mm and 34 × 150 mm) (Fig. 2A, B). The proximal end of the stent was located in the lower of the left subclavian artery and the distal end lies above the celiac trunk artery. The position of the stents was normal by arteriography. The celiac trunk artery and the superior mesenteric artery were unobstructed; and there had no internal leakage between the connections of the stents (Fig. 2C). The patient was admitted to the ward of ICU after the operation and had no severe complications. The patient was discharged on the seventh day after surgery. Patient was regularly followed up in the outpatient department of our hospital, CT examination on January 21, 2019 found that the stents were in good position and no internal leakage was observed.

## 3 Discussion

Because of residual aortic aneurysm or aortic dissection, patients with MFS usually need repeat operations after the first operation.^[12]^ The first occurrence in our case was mainly manifested as the formation of an abdominal aortic dissection and the second onset primarily manifested as Stanford type A aortic dissection. The first 2 surgeries were routine open abdominal and thoracic operations for artificial blood vessel replacement. Our patient's third surgery was primarily for residual aortic aneurysm expansion, which is consistent with the pathological characteristics in patients with MFS. Several major problems presently exist for the treatment of our patient. First, severe tissue adhesion and disappearance of normal tissue space were apparent after 2 open operations. The maximum diameter of the residual dilated aorta was 75 mm and caused thinning of the wall of the aorta. Operational risks, complications, and mortality would significantly increase with repeated open surgery. Second, if we performed open chest surgery, surgical anastomosis of the artificial vessel could be affected by the insertion of the stent into the descending aortic in the first operation. Therefore, we chose to perform TEVAR.

Currently, for thoracic and abdominal aortic lesions, intravascular stent intervention has replaced routine open heart and open abdominal surgery as the preferred treatment. Patients with MFS usually have systemic tunica media arterial lesions. Considering the safety of the anchorage area, endovascular stent implantation is generally not recommended for patients with MFS. Some studies indicate that repeat surgical intervention rates in patients undergoing endovascular stent placement are as high as 66% to 83% in the short term because of various complications.^[13]^ However, attempts at interventional therapy in patients with MFS have not been abandoned. Endovascular treatment can provide a useful adjunct or bridge to open surgical treatment in selected patients.^[7]^ Guering^[8]^ performed endovascular stent implantation in 51 patients with MFS and Stanford type B aortic dissection, revealing that 4 patients needed to repeat open operations due to complications, the hospital mortality rate was 10%, mortality during median follow-up (mean 59.6 ± 38.9 months) was 20%, and the incidence of internal leakage was 44.4%. Other studies have shown that the early mortality rate for TEVAR treatment in patients with MFS is 12.5% to 20.0%, the incidence of internal leakage is 33.3%, and the rate of reoperation with interventional therapy is approximately 33.0% to 46.6%.^[9–11]^

Reported cases show a high incidence of internal leakage after endovascular repair in patients with MFS. We consider mainly that patients with MFS have abnormal vessels, the anchorage area of the stent is not firmly fixed, and the self-expanding stent can also indirectly lead to blood vessel dilation. Our case had previously experienced 2 surgeries for ascending aorta and abdominal aortic replacement and descending aorta-nasal stent implantation; however, the vessel of this lesion was located in the residual part of the 2 operations. We believe that the upper end of the lesion was the aorta-nasal stent rather than the aorta, and it could provide a sufficient and stable anchorage area for the stent vessels. The lower end of the lesion was the anastomotic artificial vascular, and it would not cause the secondary expansion of blood vessels due to the swelling of the stent. Therefore, we decided to perform TEVAR in our patient, and postoperative imaging revealed that the stent was fixed in place and the tumor cavity disappeared.

There are still numerous problems in endovascular stent implantation in patients with MFS with high surgery risk or multiple operations. But through this case, we have a bold vision of using artificial material to fix and package the area of the residual vessel during the first surgery, which can provide an anchor area for future endovascular stent implantation and also provide a possibility for stent implantation in patients with MFS.

## Author contributions

**Conceptualization:** Ximing Qian.

**Methodology:** Ximing Qian.

**Supervision:** Ximing Qian.

**Visualization:** Ximing Qian.

**Writing – original draft:** Fan He.

**Writing – review & editing:** Huai-Dong Chen.
